# Uncovering risky behaviors of expatriate teenagers in the United Arab Emirates: A survey of tobacco use, nutrition and physical activity habits

**DOI:** 10.1186/s12889-015-2261-9

**Published:** 2015-09-23

**Authors:** Leena W. Asfour, Zachary D. Stanley, Michael Weitzman, Scott E. Sherman

**Affiliations:** New York University Abu Dhabi, P.O. Box 129188, Abu Dhabi, United Arab Emirates; Division of General Internal Medicine, New York University School of Medicine, New York, NY USA; Departments of Pediatrics and Environmental Medicine, Professor of Global Public Health, New York University School of Medicine, New York University, New York, NY USA

**Keywords:** Tobacco, Nutrition, Physical activity, UAE, Shisha, Dokha, Health education, Teenagers, “Risky”, “Behaviors”, “Gulf”, “UAE”, “Tobacco”, “Hookah”, “Medwakh”, “Expatriates”, “High school”

## Abstract

**Background:**

Tobacco use and unhealthy lifestyle habits amongst youth contribute to most major health issues in the United Arab Emirates (UAE) and worldwide. However up to date and comprehensive statistics are not available on the current behavior, experimentation and environmental influences on teenagers in the UAE’s expatriate community, who are greatly impacted by the country’s culture and environment, as well as bringing influences from their cultures of origin. Expatriates comprise a majority of the UAE population, making them an important subset of the population to study.

**Method:**

To address this gap in knowledge, a survey was conducted to collect information on tobacco use, physical activity and nutrition behaviors, anti-tobacco media/legislation effectiveness and health education gaps.

**Results:**

Our results provide a summary on each of these topics with regards to ninth grade expatriates in the UAE. We offer the first statistics on dokha use in this age group and uncover signs of underlying eating disorders.

**Conclusions:**

In conclusion, we call for a tobacco use, nutrition and physical activity intervention targeted at this age group of UAE expatriates.

## Background

In the fight against non-communicable diseases, it is well established that a few key lifestyle choices can act as profoundly effective preventive measures. These include choices regarding tobacco use, nutrition, and physical activity [1]. Habits that are formed around these behaviors develop at a young age, and may often follow a person throughout their adult life [2].

Geographical regions such as the Middle East, where tobacco use has long been an integral part of the culture, have recently experienced an even greater prevalence of tobacco use, especially among youth [3]. The number of teenagers using tobacco products, such as shisha (tobacco or non-tobacco based herbal materials smoked in a water pipe or hookah), dokha (a tobacco popular around the Persian Gulf, smoked through a midwakh pipe), and cigarettes is very high in the United Arab Emirates (UAE) [4]. Although the legal smoking age is 18 years, the most recent statistics available reveal that 12 % of males and 2.5 % of females aged 13–15 years are current cigarette smokers [5]. In this same age group 19 % of males and 10 % of females smoke shisha [5]. Dokha prevalence in this age group is unknown, although one study reports that 11.5 % of medical students in Ajman, UAE, had used dokha [6]. A study from 2010 states that of young cigarette smokers in the UAE, over 82 % of them tried their first cigarette before the age of 14 [7]. None of these statistics, however, apply solely to expatriate communities in the country. Moreover, tobacco products are cheap (approximately 2.5 USD for a pack of cigarettes) and easily accessible. Shisha smoking is considered an activity that is more socially acceptable than cigarette smoking in this region and often perceived to be less dangerous [8].

In addition to smoking, obesity is very common in the UAE. This frequently results in health complications including diabetes, hypertension and cardiovascular diseases such as myocardial infarctions and strokes . The UAE is ranked 15^th^ in the world for incidence of type II diabetes with almost 19 % of the population living with this condition [9], and the prevalence is increasing. The rapid increase in the number of type II diabetes cases in this country has been attributed to the rise of obesity and the pervasiveness of a sedentary lifestyle coupled with increased access to unhealthy diet options [10].

Not only can tobacco use and diabetes lead to life threatening diseases, but they also are part of the healthcare cost burden in the UAE and elsewhere. The UAE government spends significantly more on healthcare costs than the global average [11]. Information on the estimated costs of smoking is not available for the UAE. However, as smoking can lead to countless diseases, the costs are likely significant. With regards to diabetes, one study found that the annual direct treatment costs of diabetes without complications in Al Ain, UAE is 3.2 times per capita higher than the per capita expenditure for healthcare in the UAE overall. This number increases significantly with complications [12].

In order to put the above statistics in perspective, the UAE has a population of just over 8 million people, 89 % of which are non-nationals, according to the last census taken in 2011 by the National Bureau of Statistics [13]. The percentage of expatriates has only increased over time, and as such, are a very important group for study. Even though non-nationals represent a huge majority of the population, studies have not been conducted to ascertain their health status or their health behaviors. Our study offers new insight on a previously overlooked segment of the population that could help inform new public health interventions in the country.

The purpose of this study is to generate up-to-date and comprehensive data on tobacco use, physical activity and nutrition behaviors, and health education gaps of expatriate ninth graders in the UAE. This is especially useful in light of recent legislative efforts to lessen tobacco use. Moreover, it is important to collect data on the large expatriate community in the UAE as their lifestyle choices are also impacted by environmental and cultural factors. To address this gap in knowledge, we conducted a survey to collect information on each area mentioned above. Our results provide a summary of risky behaviors and several environmental factors that influence teenage behavior. We also offer the first statistics on dokha use in this age group and uncover signs of underlying eating disorders.

## Methods

In this case–control study, our cohort consisted of ninth grade students from five private high schools in the city of Abu Dhabi. According to the Abu Dhabi Education Council, there are 185 private schools across the entire emirate. These schools represented the most accessible populations for our study and thus were samples of convenience. Each school had a student body primarily composed of students from a North African or Middle Eastern (MENA) descent. We excluded schools whose pupils did not read and write English at a level that would allow them to answer the survey. We focused on ninth graders because the literature suggested that smoking tobacco becomes a highly relevant topic around this age. As noted earlier, 82 % of young smokers in the UAE had tried their first cigarette before the age of 14 [7]. Both parental and minor consent forms were distributed and returned before scheduling the survey. Of the classes that were tested, all students participated, with some discrepancies arising for student absences during workshop or survey dates.

We adapted the Global Youth Tobacco Survey (GYTS) from the World Health Organization (WHO), a survey that has been used in the MENA region before, to fit the cultural context of the UAE [14]. Fifty-seven questions were used from this survey to gather information related to tobacco use, including questions about environment and education. Following the GYTS question style, we added 12 questions about dokha use. The Centers for Disease Control’s Youth Risk Behavior Surveillance System (YRBSS) was used as a source for questions about nutrition and physical activity [15]. Twenty-three questions were used from this survey. In total, the survey had 92 questions.

We administered the survey to 18 gender-segregated classrooms (10 female, 8 male). The statistical software R was used to ascertain frequencies, averages and correlations.

All steps of the methodology described above were approved by the Institutional Review Board of New York University Abu Dhabi on September 3, 2013.

## Results

The sample consisted of 439 total respondents (53.5 % female). The average age of the cohort is approximately 14 years old. The average weekly disposable income of the students is 94 Dirhams, or approximately $26 US dollars. This is not surprising, as expatriates with families must meet a certain income level to apply for a residence visa and matches the trend of above average income in comparison with other rapidly developing countries in the region.

Table 1 shows how many students had experimented with tobacco products by gender and the average age at initiation of experimentation. Notably, the average age of experimentation for females was younger than males for all forms of tobacco. A breakdown of students who had used tobacco products in the last 30 days is shown in Table 2.

**Table 1:** Experimentation with Tobacco Products

Have you ever tried or experimented with _________ smoking, even one or two puffs?	Male Freq.	Average Age *(years)	Female Freq.	Average Age *(years)
Cigarettes (*n* = 204, *n* = 229)	32 %	12-13	19 %	11-12
Shisha (*n* = 203, *n* = 234)	39 %	12-13	37 %	11-12
Dokha ** (*n* = 202, *n* = 233)	20 %	13-14	7 %	11-12

*Average age refers to the age at which respondents first reported trying the tobacco product

**Dokha is a type of tobacco popular in the Persian Gulf region, which is smoked through a midwakh pipe

**Table 2:** Tobacco use patterns of students who smoked in the last 30 days

Tobacco Form	Frequency		Average number of days they smoked in last 30 days		Average usage per day	
	Males	Females	Males	Females	Males	Females
Cigarettes (*n* = 204, *n* = 231)	9.8 %	4.3 %	7	4	3 cigarettes	2 cigarettes
Shisha (*n* = 203, *n* = 234)	17.2 %	12.8 %	5	3	1 session	1 session
Dokha (*n* = 203, *n* = 232)	15.2 %	2.6 %	25	7	2-3 sessions	1 session

Possible environmental factors influencing participants’ use or non-use of tobacco products can be seen in Appendix 1: Table 4. In the past 7 days, 45 % had someone smoking in their home, in their presence at least once that week. The majority (62 %) reported that at least some of the people in their grade smoked. Forty-eight percent had at least some close friends that smoked. This data showed that a large percentage of the cohort was exposed to tobacco through many different channels.

In terms of education, approximately 50 % have not talked in class about the health effects or dangers of tobacco use or discussed why teenagers smoke. Almost 58 % had not read about the health effects of tobacco in their textbooks. Fifty two percent had not discussed the harmful effects of tobacco use with their family.

With regards to anti-tobacco messages, 45.5 % reported that they had seen anti-tobacco messages on cigarette packages, but they did not lead the students to think differently about tobacco. Around thirty percent did not see any anti-tobacco messages on cigarette packages. Almost one fourth (24.4 %) had seen anti-tobacco messages on shisha tobacco packages, but they did not lead the students to think differently about shisha.

Of students who used tobacco products, 62.3 % reported that their age did not prevent them from purchasing cigarettes. Over 51 % reported that their age did not prevent them from being served shisha. Of note, we found that disposable income was only weakly correlated with tobacco product experimentation: cigarettes (r = 0.06, *p* < 0.05), shisha (r = 0.12, *p* < 0.05), dokha (r = 0.25, *p* < 0.05).

With regard to nutrition and physical activity behaviors, students reported consuming soft drinks twice a day. They also reported on average exercising 3 days a week for 60 min per day. Interestingly 55.5 % of the cohort reports currently trying to lose weight even though only 23.5 % reported that they were slightly overweight, while another 4.6 % reported they were very overweight. Almost 70 % reported that they had received advice on how to lead a healthy lifestyle. Appendix 2: Table 5 and 6 describe in detail the cohort’s perception of their weight, their levels of physical activity and nutrition habits.

Using the questions adapted from the YRSS asking about eating habits over the last 30 days to lose or keep from gaining weight, we found that 18.8 % of students said that they had gone more than a day without eating for these purposes, 8.5 % reported using diet pills, powders, or liquids and 6.9 % reported vomiting or taking laxatives. Another 4.4 % responded that they had smoked tobacco in order to lose weight or keep from gaining weight. A breakdown of the responses by gender can be found in the Table 3.

**Table 3:** Indications of eating disorders

Survey Question	Freq. of Males Responding Yes	Freq. of Females Responding Yes
During the past 30 days, did you smoke tobacco to help you lose weight or keep from gaining weight? (*n* = 202, *n* = 232)	6.4 %	2.6 %
During the past 30 days, did you go without eating for 24 hours or more (also called fasting) to lose weight or to keep from gaining weight? (*n* = 204, *n* = 228)	15.2 %	21.3 %
During the past 30 days, did you take any diet pills, powders, or liquids without a doctor's advice to lose weight or to keep from gaining weight? (*n* = 204, *n* = 232)	6.9 %	9.8 %
During the past 30 days, did you vomit or take laxatives to lose weight or to keep from gaining weight? (*n* = 204, *n* = 231)	6.4 %	7.2 %

## Discussion

This is the first study that we are aware of to provide survey results of teenage risk taking behaviors among expatriate school aged children in the Persian Gulf region. Unexpectedly, our survey elucidated an underlying eating disorder issue with a high percentage of students engaging in vomiting, fasting, using tobacco or laxatives and other chemicals to lose weight. Moreover, a higher percentage of people are trying to lose weight than the percentage of people who reported being overweight, which may be another indicator of a growing eating disorder issue. This risky behavior, though unanticipated, must be addressed.

On the other side of the spectrum, we were unsurprised to find that our cohort consumes a large amount of sugary drinks per day and does not fulfill the recommended exercise time of 60 min per day as shown in Appendix 3: Table 6. Both these behaviors increase the students’ risk for diabetes.

Our findings regarding cigarette use and shisha use are consistent with other studies, with higher percentages of female use. A puzzling statistic is the experimentation age for males versus females. Males first tried cigarettes and shisha between the ages of 12 and 13 years and dokha between 13 and 14 years. Females, however, first tried all three tobacco products between 11 and 12 years. Future studies should explore reasons for this discrepancy, as the frequency of females who smoke in this age group appears to be increasing.

Perhaps the more pressing issue is the frequency of dokha use. Fifteen percent of males responded that they use dokha, making it more frequent than cigarette use (10 %) and almost as high as shisha use (17 %). On average, males who do smoke dokha use it two to three times a day almost every day. While research on the biological effects of dokha is still developing, this data supports the notion that dokha may be highly addictive. The growing popularity of this unregulated drug is not only a concern for the Persian Gulf region, but also for the international community as students from the region increasingly seek foreign education.

Furthermore, these results indicate that anti-tobacco messages and the law against underage smoking have been ineffective. This along with the consistent tobacco use frequencies, point to the fact that the legislative efforts of the last few years have not reached the younger generation, which is the age group most vulnerable to addiction.

The most effective anti-tobacco campaigns have typically involved increasing prices of tobacco products [16]. It is evident from these data that disposable income has little bearing on tobacco accessibility due to how cheap the products are. As the UAE does not collect taxes on any goods, it is unlikely that a traditional tax will be acceptable. Feasible ways to incorporate the benefits of a price increase should be examined.

An unexplored option is health education. Overall, our data reveals that there is a major gap in health education both at home and in school. Because a large percentage of this group is exposed to tobacco use by their peers, family members, and/or other people in their home, it is important that tobacco use interventions are far-reaching and effective. Schools are ideal for comprehensive, uniform health education interventions as the information not only reaches the population at an age that has the potential to impact future behavioral decisions, but also functions as a way to easily disseminate information to a large audience.

As with all survey research, there are several biases that should be taken into consideration when analyzing the data. One of the most powerful biases is the social desirability bias. Even though respondents were assured that their answers would remain completely anonymous and detached from any identifying information, the cultural setting of the UAE may prevent students from reporting taboo behaviors. As tobacco policies in the country and schools become better enforced, students may become less willing to open up about their experiences for fear they will be punished.

Although the survey directions specified that “tobacco smoking” referred to dokha, shisha and cigarette use, we observed that most students associated the term with cigarette use only. This may have an influence on how students responded to questions with this terminology.

Despite the limitations, these data provide vitally important new information about a large group of individuals in the UAE that has not previously been the focus of health related research or special attention to the health of its members.

## Conclusions

Our results provide a summary on behaviors related to physical activity, nutrition and tobacco use in expatriate ninth graders in the UAE. Statistics on dokha use and signs of underlying eating disorders are especially concerning. We call for a tobacco use, nutrition and physical activity intervention targeted at this age group of UAE expatriates.

**Table 4:** Tobacco environment (school, home, peers, media, access)

Survey Question	Answer Choice	Frequency
Do your parents smoke tobacco? (*n* = 438)	None	64.8 %
	Both	8.9 %
	Father only	19.2 %
	Mother only	1.6 %
	Don't know	5.5 %
How often do you see your brother/sister smoking in your home? (*n* = 434)	Don't have/don't see this person	11.1 %
	About every day	3.9 %
	Sometimes	9.7 %
	Never	75.3 %
How often do you see other people smoking in your home? (*n* = 434)	Don't have/don't see this person	7.1 %
	About every day	15.9 %
	Sometimes	42.6 %
	Never	34.3 %
About how many students in your grade smoke? (*n* = 434)	Most of them	7.6 %
	About half of them	11.1 %
	Some of them	42.9 %
	None of them	38.5 %
Do any of your closest friends smoke tobacco? (*n* = 435)	All of them	3.2 %
	Most of them	5.7 %
	Some of them	38.6 %
	None of them	52.4 %
During the past 12 months, did you read in your school texts or books about the health effects of tobacco? (*n* = 434)	Yes	38.9 %
	No	57.8 %
	I do not have school texts or books	3.2 %
During the past 12 months, were you taught in any of your classes about the dangers of tobacco use? (*n* = 437)	Yes	33.2 %
	No	45.5 %
	I don’t know	21.3 %
During the past 12 months, did you discuss in any of your classes the reason why people your age use tobacco? (*n* = 432)	Yes	21.1 %
	No	52.3 %
	Not sure	26.6 %
Has anyone in your family discussed the harmful effects of smoking tobacco with you? (*n* = 435)	Yes	52.4 %
	No	47.6 %
During the past 12 months, were you taught in any of your classes about the effects of using tobacco like it makes your teeth yellow, causes wrinkles, or makes you smell bad? (*n* = 434)	Yes	37.1 %
	No	41.5 %
	Not sure	21.4 %
Have you ever received help or advice to help you stop smoking? (*n* = 437)	I have never smoked	75.7 %
	Yes, from a program or professional	0.0 %
	Yes, from a friend	5.0 %
	Yes, from a family member	4.1 %
	Yes, from both programs or professionals and from friends or family members	2.5 %
	No	12.6 %
During the past 30 days, did you see or hear any anti-tobacco media messages on television, radio, internet, billboards, posters, newspapers, magazines, or movies? (*n* = 435)	Yes	56.0 %
	No	44.0 %
During the past 30 days, did you see or hear any anti-tobacco messages at sports events, fairs, concerts, or community events, or social gatherings? (*n* = 435)	I did not go to sports events, fairs, concerts, or community events, or social gatherings in the past 30 days	33.6 %
	Yes	25.7 %
	No	40.7 %
During the past 30 days, did you see any health warnings on cigarette packages? (*n* = 435)	Yes, but I didn’t think much of them	45.5 %
	Yes, and they led me to think about quitting smoking or not starting smoking	23.7 %
	No	30.8 %
During the past 30 days, did you see any health warnings on shisha tobacco packages? (*n* = 435)	Yes, but I didn’t think much of them	24.4 %
	Yes, and they led me to think about quitting shisha smoking or not starting shisha smoking	12.2 %
	No	63.4 %
*The last time you smoked cigarettes during the past 30 days, how did you get them? (*n* = 438)	I bought them in a store or shop	35.1 %
	I bought them from a vending machine	5.4 %
	I got them from someone else	54.1 %
	I got them some other way	5.4 %
*The last time you smoked shisha during the past 30 days, where did you smoke it? (*n* = 434)	At home	26.0 %
	At a coffee shop	46.8 %
	At a restaurant	9.1 %
	Other	18.1 %
*The last time you smoked dokha during the past 30 days, where did you smoke it? (*n* = 434)	At home	10.0 %
	At a coffee shop	15.0 %
	At a restaurant	5.0 %
	At a bar or club	2.5 %
	Other	67.5 %
*During the past 30 days, did anyone refuse to sell you cigarettes because of your age? (*n* = 436)	Yes, someone refused to sell me cigarettes because of my age	38.7 %
	No, my age did not keep me from buying cigarettes	62.3 %
*During the past 30 days, did anyone refuse to serve you shisha because of your age? (*n* = 435)	Yes, someone refused to serve me shisha because of my age	48.6 %
	No, my age did not keep me from being served shisha	51.4 %

*Of students who smoked in the last 30 days

**Table 5:** Weight perception and access to support for living a healthy lifestyle

Survey Question	Answer Choice	Frequency
How do you describe your weight? (*n* = 434)	Very underweight	6.0 %
	Slightly underweight	13.8 %
	About the right weight	52.1 %
	Slightly overweight	23.5 %
	Very overweight	4.6 %
Which of the following are you trying to do about your weight? (*n* = 436)	Lose weight	55.0 %
	Gain weight	9.6 %
	Stay the same weight	20.6 %
	I am not trying to do anything about my weight	14.7 %
Have you ever received help or advice to help you maintain a healthy lifestyle? (*n* = 430)	Yes	69.5 %
	No	30.5 %
During the past 12 months, on how many sports teams did you play? (Count any teams run by your school or community groups.) (*n* = 429)	0 teams	34.0 %
	1 team	34.3 %
	2 teams	15.9 %
	3 or more teams	15.9 %
During the past 7 days, on how many days were you physically active for a total of at least 60 minutes per day? (Add up all the time you spent in any kind of physical activity that increased your heart rate and made you breathe hard some of the time.) (*n* = 428)	0 days	16.4 %
	1 day	12.6 %
	2 days	14.5 %
	3 days	15.2 %
	4 days	11.9 %
	5 days	10.5 %
	6 days	3.3 %
	7 days	15.7 %

**Table 6:** Description of current nutrition and physical activity levels

Survey Question	Response Average
During the past 7 days, how many times did you drink 100 % fruit juices such as orange juice, apple juice, or grape juice? (Do not count fruit-flavored drinks.)	4-6 times during the past 7 days
During the past 7 days, how many times did you eat fruit? (Do not count fruit juice.)	1 time per day
During the past 7 days, how many times did you eat green salad?	4-6 times during the past 7 days
During the past 7 days, how many times did you eat other vegetables? (Do not count green salad)	4-6 times during the past 7 days
During the past 7 days, how many times did you drink a can, bottle, or glass of soda or pop, such as Coke, Pepsi, or Sprite? (Do not count diet soda or diet pop.)	2 times per day
During the past 7 days, how many glasses of milk did you drink? (Count the milk you drank in a glass or cup, from a carton, or with cereal. Count the half pint of milk served at school as equal to one glass.)	4-6 glasses during the past 7 days
During the past 7 days, on how many days did you eat breakfast?	5 days
On an average school day, how many hours do you watch TV?	1-2 hours
On an average school day, how many hours do you play video or computer games or use a computer for something that is not school work? (Count time spent on things such as Xbox, PlayStation, an iPod, an iPad or other tablet, a smartphone, YouTube, Facebook or other social networking tools, and the Internet.)	2 hours
In an average week when you are in school, on how many days do you go to physical education (PE) classes?	1-2 days
